# Epidemiology of Lower Extremity Amputations in the United States: An Analysis of the Global Burden of Disease Database From 1990 to 2019

**DOI:** 10.7759/cureus.95188

**Published:** 2025-10-22

**Authors:** Peter B Spencer, Cameron J Sabet, Gabrielle Dykhouse, Taylor J Manes, Phillip C McKegg, Ambrose Loc T Ngo, Ayah Ibrahim, Jack Weick

**Affiliations:** 1 Orthopedic Surgery, Ohio University Heritage College of Osteopathic Medicine, Cleveland, USA; 2 Orthopedic Surgery, Georgetown University School of Medicine, Washington, DC, USA; 3 Orthopedic Surgery, Cornell University School of Medicine, New York, USA; 4 Orthopedic Surgery, OhioHealth Doctors Hospital, Columbus, USA; 5 Orthopedic Surgery, Henry Ford Hospital, Detroit, USA; 6 Orthopedic Surgery, Kansas City University College of Osteopathic Medicine, Joplin, USA; 7 Orthopedic Surgery, Burrell College of Osteopathic Medicine, Las Cruces, USA; 8 Orthopedic Surgery, Grant Medical Center, Columbus, USA

**Keywords:** amputation, epidemiology, global burden of disease, lower extremity, north america

## Abstract

Background: Lower extremity amputations (LEAs) are medically, psychologically, and functionally devastating. The objective of our study was to evaluate region- and sex-specific differences of unilateral and bilateral LEAs across the United States (US) from 1990 to 2019.

Methods: The Global Burden of Disease database was used to analyze years lived with disability (YLDs), prevalence, and incidence rates per 100,000 people for LEAs in the US from 1990 to 2019. Data were stratified into four US Census Bureau-defined regions: Northeast, Midwest, South, and West. Differences between regions and sexes were assessed, with statistical significance defined as p < 0.05.

Results: From 1990 to 2019, the US experienced an overall decrease in YLDs (33.07%), incidence (22.11%), and prevalence (29.93%) of bilateral LEAs. Unilateral LEAs saw a decrease in YLDs (21.43%) and prevalence (15.61%), but an increase in incidence (9.05%). Men surpassed women in YLDs, incidence, and prevalence of unilateral and bilateral LEAs in all regions. The West had the highest incidence and prevalence of both bilateral and unilateral LEAs from 1990 to 2019. By 2019, the South had the lowest incidence, and the Northeast had the lowest YLDs and prevalence of bilateral and unilateral LEAs.

Conclusions: From 1990 to 2019, the US experienced decreases in YLDs, prevalence, and incidence of unilateral and bilateral LEAs, except for an increased incidence of unilateral LEAs. Men experienced higher rates than women across each region. The West generally had the highest overall rates. These trends highlight sex-specific and regional disparities of LEAs.

## Introduction

Each year, roughly 150,000 patients undergo lower extremity amputations (LEAs) in the United States (US). The main causes are diabetes mellitus, peripheral vascular disease, neuropathy, and trauma, accounting for significant morbidity and healthcare costs [1]. Epidemiological studies previously have provided a valuable view of the awareness, causes, and consequences of LEAs, which can inform interventions to improve patient outcomes [2-4].

Published reports suggest that LEA rates vary significantly by geography and the population studied. For instance, Moxey et al. reported significant geographic variation in LEA incidence globally [2]. Zhang et al. used the Global Burden of Disease (GBD) 2016 database to analyze LEAs related to diabetes [5]. In the US specifically, the Global Lower Extremity Amputation Study Group reported that the Navajo Nation was the leading group in terms of nationwide prevalence of LEAs [6].

Despite the prevalence of these amputations across the US, however, there are no studies specifically examining regional variations in LEAs over time specifically focused on the US. For example, while studying global patterns is important, it does not fully account for the nuanced differences in how LEAs present, are treated, and resolve under the influences of the US healthcare system. Hughes et al. have previously demonstrated variations in LEAs across the European Union, but no similar study has been performed across the US [7].

Given the gap of US-based LEA prevalence studies in the literature, the objective of this study was to define a detailed temporal analysis of the epidemiology of LEAs in the US between 1990 and 2019 using the 2021 GBD database. Specifically, this study explored annual trends of incidence, prevalence, and years lived with disability (YLDs) of LEAs during this period. These findings can help in the identification of certain trends by analyzing the various factors contributing to the development of LEAs and how orthopedic leaders can mitigate the impact of LEAs moving forward. Apart from outlining regional variations, the findings could also hold significant implications for clinical practices across various patient demographics.

This study was previously presented by the lead author as a virtual abstract presentation at the 2024 Student American Osteopathic Academy of Orthopedics Annual Fall Meeting on October 25, 2024.

## Materials and methods

Study design and data sources

This ecological study utilized the GBD dataset, developed by the Institute for Health Metrics and Evaluation [8]. The GBD dataset includes epidemiological data on 369 diseases and injuries across 204 countries from 1990 to 2019. It draws from a variety of sources including administrative records, census data, demographic surveys, geospatial data, and modeled data to provide estimates and projections [8]. The methods for developing the GBD dataset are well-documented, and the disease burden estimates have been validated [9-11]. The sampling technique of this study draws upon all the relevant data in the GBD dataset. The GBD dataset sampling technique has been previously described [8]. Data regarding unilateral and bilateral LEAs were obtained from the GBD dataset using keywords and disease categories. After the data were obtained, it was stratified into four regions-Northeast, Midwest, South, and West-based on the US Census Bureau definitions [12]. Data analysis was then carried out as described below and interpreted by the authors. There were no specific inclusion or exclusion criteria as part of this study design, as all the data were obtained from a public dataset. Any inclusion/exclusion criteria described by the GBD dataset would carry over into this study, as this was the data source.

Outcomes

The primary outcomes of interest were YLDs, incidence, and prevalence of LEAs. According to the World Health Organization, a YLD represents "one full year of healthy life lost due to disability or ill-health” [13]. Age-standardized rates of YLDs, prevalence, and incidence per 100,000 people were collected for both men and women, for each state and the entire US population. Institutional review board approval was not required as the study utilized de-identified, publicly accessible data.

Statistical analysis

The statistical analysis methods applied in this study have been previously described and published [14]. An analysis of variance (ANOVA) was conducted to evaluate the need for multiple corrections across all measures. Bartlett’s test assessed the dataset's variance to determine if it was equal or unequal. For datasets with unequal variance, Welch’s ANOVA was performed to examine regional differences in YLDs, prevalence, and incidence, followed by a Games-Howell post hoc test for multiple comparisons. For datasets with equal variance, Tukey’s post hoc analysis was used. Additionally, independent t-tests compared the mean YLDs, prevalence, and incidence rates between men and women by region and for the entire US. Statistical significance was set at p < 0.05. All analyses were performed using IBM SPSS Version 29 (IBM Corp., Armonk, NY, US).

## Results

United States

**Figure 1:**
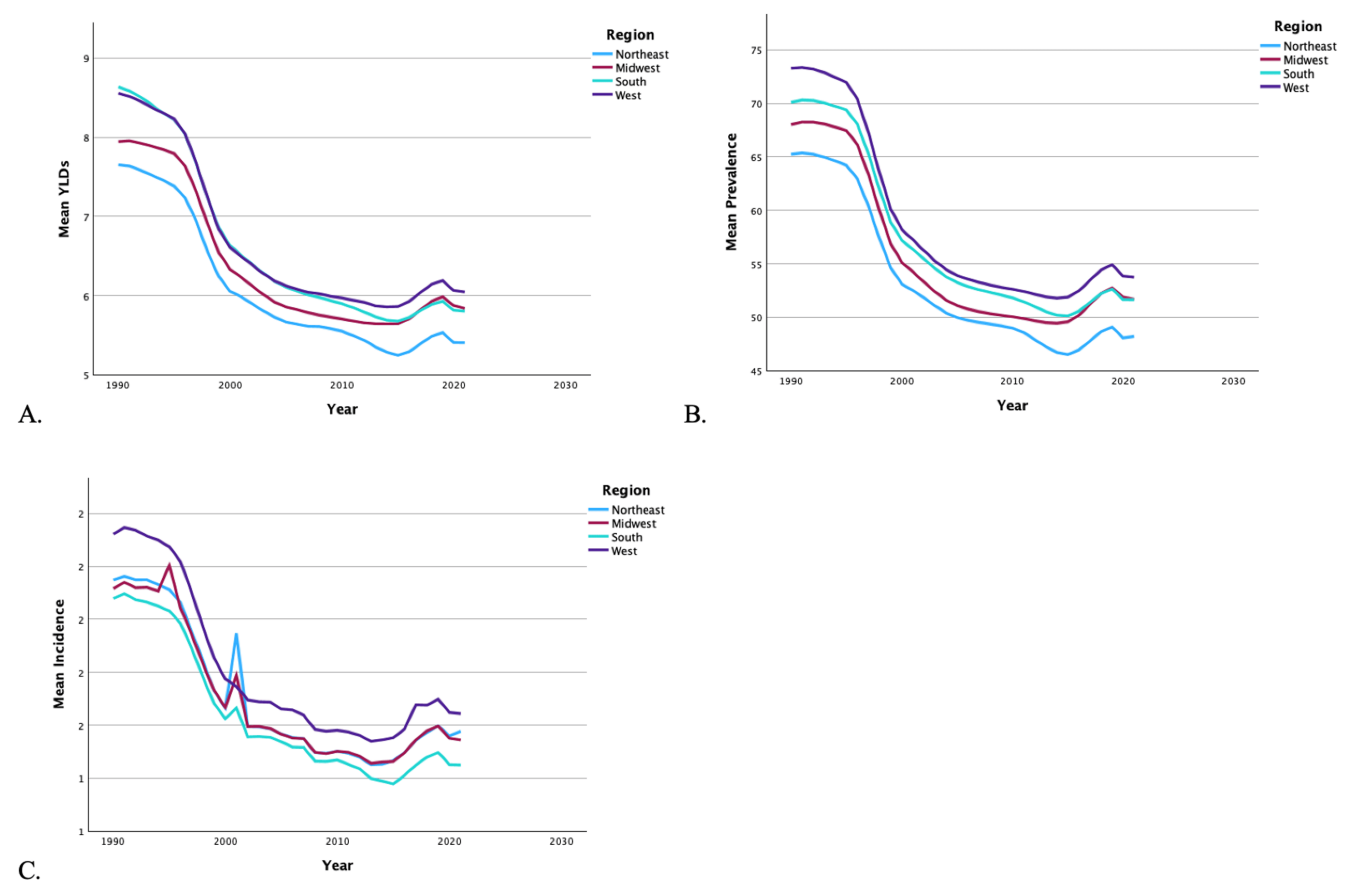
Temporal trends of YLDs, prevalence, and incidence for bilateral lower extremity amputations by region. Graphs represent the temporal trends from 1990 to 2019 in mean YLDs (A), mean prevalence (B), and mean incidence (C) of bilateral lower extremity amputations, categorized by regions: Northeast, Midwest, South, and West. YLDs: years lived with disability

**Figure 2:**
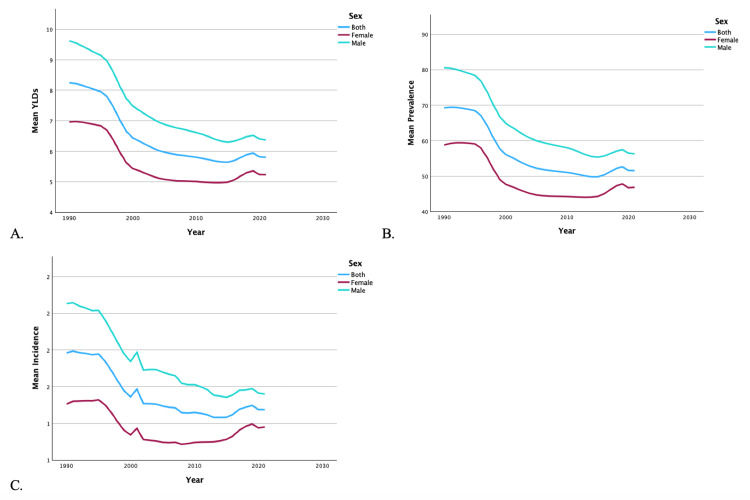
Temporal trends of YLDs, prevalence, and incidence for bilateral lower extremity amputations by sex. Graphs illustrate temporal trends from 1990 to 2019 in mean YLDs (A), mean prevalence (B), and mean incidence (C) of bilateral lower extremity amputations, stratified by sex. YLDs: years lived with disability

From 1990 to 2019, the US saw a 33.07% decrease in YLDs, a 22.11% decrease in incidence, and a 29.93% decrease in prevalence of bilateral LEAs. Regardless of region (Figures 1A-1C) or sex (Figures 2A-2C), there was a decrease in overall mean YLDs, incidence, and prevalence of bilateral LEA from 1990 to 2019. The mean YLDs (7.13 vs. 5.37), incidence (1.68 vs. 1.32), and prevalence (62.13 vs. 47.25) of bilateral LEA were significantly higher in men than women (p < 0.001). Women never surpassed men in YLDs, incidence, and prevalence of bilateral LEA in the time period.

**Figure 3:**
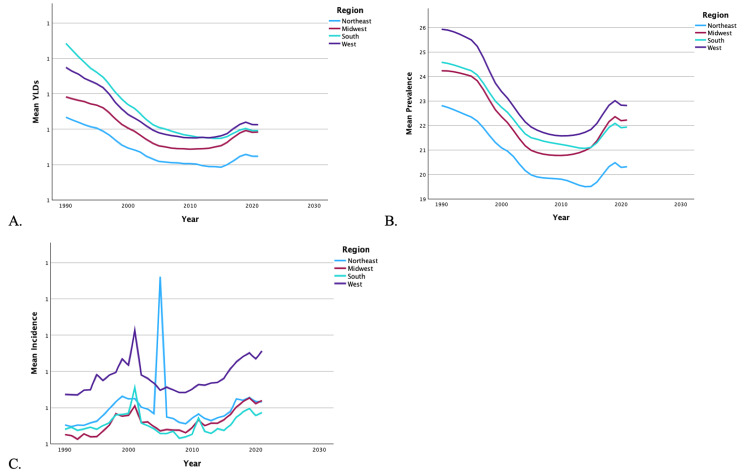
Temporal trends of YLDs, prevalence, and incidence for unilateral lower extremity amputations by region. The graphs present the temporal trends from 1990 to 2019 in mean YLDs (A), mean prevalence (B), and mean incidence (C) of unilateral lower extremity amputations, categorized by regions: Northeast, Midwest, South, and West. YLD: years lived with disability

**Figure 4:**
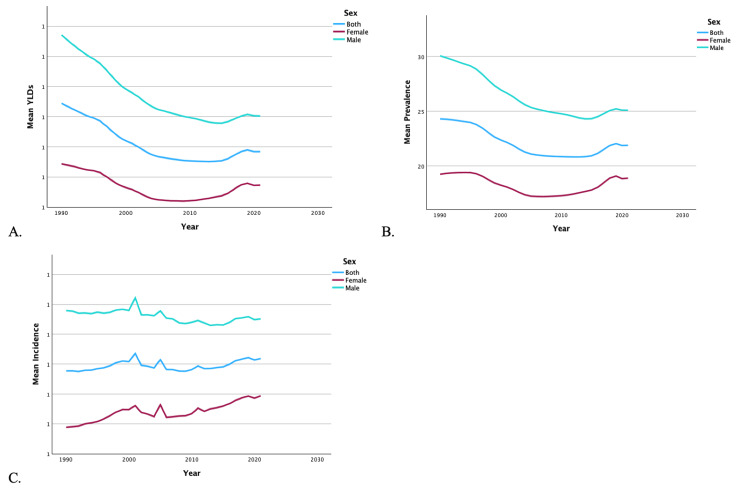
Temporal trends of YLDs, prevalence, and incidence for unilateral lower extremity amputation by sex. These figures illustrate temporal trends from 1990 to 2019 in mean YLDs (A), mean prevalence (B), and mean incidence (C) of unilateral lower extremity amputations, stratified by sex. YLDs: years lived with disability

From 1990 to 2019, the US also saw a 21.43% decrease in YLDs, a 9.05% increase in incidence, and a 15.61% decrease in prevalence of unilateral LEA. Regardless of region (Figures 3A-3C) or sex (Figures 4A-4C), there was a decrease in overall mean YLDs and prevalence of unilateral LEA from 1990 to 2019. Regardless of region, there was an increase in the mean incidence of LEA, while men experienced a decrease and women experienced an increase in incidence. The mean YLDs (0.93 vs. 0.64), incidence (1.11 vs. 0.80), and prevalence (25.35 vs. 17.53) of unilateral LEA were significantly higher in men than women (p < 0.001). Women never surpassed men in YLDs, incidence, or prevalence of unilateral LEAs between 1990 and 2019.

Data by region

Regional analysis of bilateral LEA demonstrated that the Southern region reported the highest overall mean YLDs in 1990, but was overtaken by the Western region in 2006 and Midwestern region by 2016. The Western region experienced the highest mean incidence from 1990 to 2019, except when the Northeast and Midwestern regions overtook it in 2001. The Western region also reported the highest overall mean prevalence during this time period. By 2019, the Southern region had the lowest mean incidence of all regions. The Northeast experienced the lowest rates of YLDs and prevalence of bilateral LEA, while the Southern region had the lowest mean incidence from 1990 to 2019. Men were more likely to experience greater YLDs, incidence, and prevalence of bilateral LEA in each of the regions compared to women (p < 0.001). A summary of the temporal trends of YLD, incidence, and prevalence for each region is shown in Figures 1A-1C.

Regional analysis of unilateral LEA demonstrated that the Southern region had the highest overall mean YLDs in 1990 but was overtaken by the Western region around 2013. The Western region had the highest incidence of unilateral LEA from 1990 to 2019, except in 2016, when the Northeastern region spiked. The Western region also experienced the highest prevalence during the time period. By 2019, the Southern region had the lowest mean incidence of all regions. The Northeast experienced the lowest rates of YLDs and prevalence of unilateral LEA from 1990 to 2019. The Midwest had the lowest incidence in 1990, but by 2012, the Southern region had dropped to the lowest rates. Men were more likely to experience higher YLDs, incidence, and prevalence of unilateral LEAs in each of the regions compared to women (p < 0.001). A summary of the temporal trends of YLD, incidence, and prevalence for each region can be found in Figures 3A-3C.

Data by state

For bilateral LEAs, the District of Columbia experienced the highest decrease in rates of YLDs (50.70%) from 1990 to 2019. Idaho had the highest decrease in incidence (25.69%), and Illinois had the highest decrease in prevalence (32.45%). From 1990 to 2019, North Dakota experienced the lowest decrease in YLDs (16.98%) and prevalence (16.14%), while Oklahoma had the lowest decrease in incidence (7.58%) of bilateral LEA. None of the states experienced an increase in YLDs, incidence, or prevalence from 1990 to 2019. A summary of the percent change from 1990 to 2019 for all outcome measures in all states can be found in Table 1.

**Table 1:** Percent change from 1990 to 2019 of YLDs, prevalence, and incidence for bilateral LEAs. YLDs: years lived with disability; LEAs: lower extremity amputations

State	YLDs 1990	YLDs 2021	% change	Prevalence 1990	Prevalence 2021	% change	Incidence 1990	Incidence 2021	% change
Alabama	9.29	6.02	-35.17	72.12	53.58	-25.71	1.85	1.51	-18.49
Alaska	10.07	6.63	-34.19	82.86	58.93	-28.88	1.98	1.68	-15.19
Arizona	8.38	6.18	-26.21	73.56	55.07	-25.14	2.13	1.68	-21.29
Arkansas	9.58	6.70	-30.03	77.31	59.60	-22.91	1.73	1.32	-23.70
California	8.18	5.32	-34.95	67.36	47.00	-30.22	1.94	1.61	-17.23
Colorado	8.49	6.35	-25.18	74.36	56.54	-23.98	1.94	1.67	-13.95
Connecticut	7.28	5.10	-29.89	63.62	45.26	-28.86	1.75	1.49	-14.68
Delaware	7.89	5.36	-32.08	66.18	47.72	-27.90	1.62	1.28	-20.79
District of Columbia	11.67	5.75	-50.70	66.83	49.83	-25.44	1.84	1.47	-19.88
Florida	8.12	5.80	-28.56	71.24	51.88	-27.18	1.85	1.50	-18.75
Georgia	9.14	5.54	-39.35	69.07	49.38	-28.50	1.69	1.34	-20.68
Hawaii	7.29	5.49	-24.68	63.84	48.35	-24.26	1.79	1.41	-21.11
Idaho	8.22	5.85	-28.81	72.31	52.07	-27.99	1.76	1.30	-25.69
Illinois	8.19	5.22	-36.34	68.47	46.25	-32.45	1.64	1.36	-17.28
Indiana	7.53	5.44	-27.74	66.22	48.69	-26.48	1.68	1.38	-18.16
Iowa	7.48	5.79	-22.63	65.53	51.42	-21.54	1.83	1.53	-16.20
Kansas	7.77	5.87	-24.51	68.20	52.05	-23.68	1.69	1.49	-12.19
Kentucky	8.01	5.92	-26.09	70.54	53.01	-24.84	1.79	1.47	-18.26
Louisiana	8.77	5.83	-33.43	69.89	52.18	-25.33	1.70	1.42	-16.56
Maine	7.50	5.71	-23.97	65.89	50.69	-23.06	1.69	1.45	-13.98
Maryland	8.14	5.86	-28.07	67.85	52.01	-23.34	1.75	1.49	-14.77
Massachusetts	7.55	5.70	-24.48	66.23	50.80	-23.30	1.62	1.33	-17.50
Michigan	7.63	5.28	-30.73	63.53	47.24	-25.64	1.74	1.46	-16.03
Minnesota	7.89	5.75	-27.03	69.32	50.99	-26.45	1.80	1.50	-16.60
Mississippi	9.69	6.44	-33.58	71.72	54.37	-24.19	1.91	1.69	-11.29
Missouri	8.32	5.99	-27.96	72.46	53.32	-26.42	1.85	1.55	-15.95
Montana	8.48	6.68	-21.28	74.39	59.32	-20.26	1.77	1.50	-15.17
Nebraska	7.84	5.90	-24.79	68.73	52.44	-23.70	1.55	1.23	-20.78
Nevada	9.32	6.16	-33.92	77.10	54.73	-29.02	1.98	1.51	-23.61
New Hampshire	7.49	5.69	-24.06	65.97	50.61	-23.28	1.87	1.53	-18.18
New Jersey	6.93	4.99	-28.10	60.80	44.47	-26.85	2.06	1.73	-15.75
New Mexico	9.94	6.55	-34.06	78.90	58.72	-25.58	1.69	1.44	-15.02
New York	7.70	4.91	-36.22	62.95	43.90	-30.26	1.61	1.21	-24.81
North Carolina	8.40	5.49	-34.66	67.93	48.93	-27.98	1.68	1.40	-16.72
North Dakota	7.47	6.20	-16.98	65.60	55.02	-16.14	1.76	1.42	-19.65
Ohio	7.47	5.43	-27.33	65.14	48.44	-25.63	1.91	1.61	-15.42
Oklahoma	8.24	6.36	-22.85	71.80	56.96	-20.67	1.67	1.54	-7.58
Oregon	8.23	5.69	-30.78	72.29	50.97	-29.50	1.87	1.49	-20.69
Pennsylvania	7.30	5.30	-27.41	64.01	47.52	-25.77	1.74	1.44	-17.30
Rhode Island	7.45	5.35	-28.22	65.26	47.60	-27.05	1.64	1.36	-17.04
South Carolina	9.15	5.67	-38.09	68.07	50.77	-25.42	1.84	1.69	-8.23
South Dakota	8.13	6.68	-17.80	71.31	59.45	-16.63	1.73	1.44	-16.65
Tennessee	8.58	5.76	-32.90	69.68	51.47	-26.14	1.68	1.37	-18.76
Texas	8.53	5.59	-34.50	72.63	49.72	-31.54	1.79	1.49	-16.73
Utah	7.65	5.59	-26.97	67.16	49.95	-25.63	1.81	1.54	-14.70
Vermont	8.00	5.93	-25.85	69.85	52.68	-24.58	1.88	1.42	-24.72
Virginia	7.54	5.40	-28.32	65.85	47.91	-27.24	1.70	1.37	-19.72
Washington	7.96	5.41	-32.11	69.66	48.02	-31.06	1.79	1.60	-10.33
West Virginia	8.20	5.82	-29.00	67.89	52.26	-23.03	1.80	1.41	-21.42
Wisconsin	7.88	6.10	-22.59	69.03	54.28	-21.38	1.91	1.62	-15.30
Wyoming	8.52	6.44	-24.46	74.70	57.19	-23.44	1.73	1.52	-12.16

In terms of unilateral LEA, the District of Columbia experienced the highest decrease in the rates of YLDs (50.56%), Arizona had the greatest drop in incidence (8.05%), and Illinois had the highest decrease in prevalence (17.53%) from 1990 to 2019. North Dakota experienced the lowest decrease in the rates of YLDs (2.95%) and prevalence (1.76%), while Nevada had the lowest decrease in unilateral LEA incidence rates (0.38%). Some states experienced an increase and others had a decrease in the rates of incidence of unilateral LEA. The highest increase was in Washington (17.17%), and the lowest was in Alabama (0.55%). None of the states experienced an increase in YLDs or prevalence from 1990 to 2019. A summary of the percent change from 1990 to 2019 for all outcome measures in all states can be found in Table 2.

**Table 2:** Percent change from 1990 to 2019 of YLDs, prevalence, and incidence for unilateral LEAs. YLDs: years lived with disability; LEAs: lower extremity amputations

State	YLDs 1990	YLDs 2021	% change	Prevalence 1990	Prevalence 2021	% change	Incidence 1990	Incidence 2021	% change
Alabama	1.13	0.82	-26.95	25.64	22.93	-10.57	1.00	1.01	0.55
Alaska	1.21	0.89	-26.21	29.93	24.84	-17.01	1.07	1.18	9.78
Arizona	0.92	0.83	-9.74	25.38	23.21	-8.55	1.24	1.14	-8.05
Arkansas	1.10	0.87	-20.86	26.35	24.42	-7.33	0.88	0.91	3.32
California	0.90	0.69	-23.67	22.52	19.08	-15.26	1.03	1.10	6.75
Colorado	0.93	0.86	-7.75	25.75	23.90	-7.18	1.05	1.12	6.64
Connecticut	0.81	0.70	-14.13	22.30	19.45	-12.79	0.96	0.99	3.73
Delaware	0.89	0.70	-21.20	22.84	19.56	-14.38	0.92	0.88	-4.33
District of Columbia	1.43	0.71	-50.56	20.86	19.06	-8.66	0.98	0.96	-1.75
Florida	0.88	0.77	-11.91	24.27	21.63	-10.89	0.88	0.85	-3.57
Georgia	1.13	0.75	-33.41	24.62	21.04	-14.52	0.90	0.91	1.11
Hawaii	0.81	0.72	-11.67	22.37	19.83	-11.37	0.85	0.90	6.49
Idaho	0.94	0.80	-14.65	25.92	22.50	-13.19	1.03	1.06	2.66
Illinois	0.93	0.70	-24.10	23.62	19.48	-17.53	0.93	0.94	1.10
Indiana	0.85	0.74	-13.23	23.36	20.65	-11.61	0.95	0.89	-6.14
Iowa	0.85	0.81	-5.82	23.53	22.45	-4.59	0.96	1.07	11.21
Kansas	0.89	0.81	-8.50	24.50	22.74	-7.18	0.94	1.02	8.70
Kentucky	0.92	0.84	-9.56	25.46	23.35	-8.30	0.97	1.06	8.55
Louisiana	1.05	0.81	-22.20	24.71	22.84	-7.59	1.02	1.07	5.01
Maine	0.83	0.77	-8.09	22.99	21.45	-6.71	0.96	0.97	1.64
Maryland	0.88	0.73	-17.43	22.34	20.30	-9.13	0.90	0.94	4.39
Massachusetts	0.81	0.73	-10.80	22.37	20.30	-9.28	1.00	1.04	4.35
Michigan	0.85	0.70	-18.52	21.51	19.53	-9.22	1.03	1.09	5.86
Minnesota	0.91	0.81	-10.93	25.16	22.69	-9.81	0.93	0.97	4.67
Mississippi	1.20	0.93	-22.42	25.59	23.84	-6.84	0.86	0.90	5.16
Missouri	0.93	0.82	-12.31	25.44	22.85	-10.19	1.06	1.15	8.45
Montana	1.01	0.92	-9.53	27.94	25.70	-8.02	1.05	1.13	7.87
Nebraska	0.89	0.80	-9.45	24.44	22.42	-8.26	0.95	1.02	7.83
Nevada	1.05	0.78	-25.56	26.30	21.80	-17.12	0.83	0.83	-0.38
New Hampshire	0.83	0.76	-9.19	23.01	21.28	-7.53	1.00	1.05	5.00
New Jersey	0.76	0.65	-13.76	20.64	18.18	-11.92	1.05	0.98	-7.28
New Mexico	1.15	0.88	-23.21	26.93	24.62	-8.57	1.10	1.19	8.02
New York	0.85	0.63	-25.59	20.77	17.63	-15.11	0.95	1.04	9.21
North Carolina	0.99	0.75	-23.68	23.99	21.03	-12.32	0.83	0.81	-2.53
North Dakota	0.85	0.83	-2.85	23.53	23.12	-1.76	0.94	0.98	4.78
Ohio	0.84	0.75	-11.25	23.08	20.93	-9.33	0.92	0.96	4.84
Oklahoma	0.94	0.88	-6.03	25.63	24.81	-3.19	0.97	1.03	6.26
Oregon	0.96	0.80	-15.80	26.21	22.60	-13.78	1.04	1.15	10.78
Pennsylvania	0.82	0.73	-10.61	22.56	20.51	-9.09	0.95	0.98	2.80
Rhode Island	0.82	0.71	-13.23	22.34	19.96	-10.66	1.05	1.09	3.26
South Carolina	1.15	0.78	-31.86	24.31	21.76	-10.46	0.90	0.96	6.35
South Dakota	0.92	0.89	-3.91	25.45	24.64	-3.16	1.03	1.13	10.15
Tennessee	1.03	0.80	-22.47	25.03	22.51	-10.07	0.99	1.04	5.09
Texas	0.97	0.77	-21.09	25.60	21.51	-16.00	1.04	1.12	7.90
Utah	0.88	0.77	-12.46	24.26	21.57	-11.07	0.98	1.05	6.90
Vermont	0.91	0.82	-9.31	24.89	22.89	-8.00	0.95	0.97	1.57
Virginia	0.86	0.74	-13.53	23.58	20.71	-12.19	1.02	0.99	-3.37
Washington	0.89	0.75	-15.58	24.37	20.88	-14.31	1.00	1.17	17.17
West Virginia	0.96	0.80	-15.95	23.94	22.70	-5.17	1.00	1.02	2.50
Wisconsin	0.87	0.83	-4.82	24.20	23.39	-3.35	1.06	1.10	3.73
Wyoming	0.98	0.87	-10.91	26.80	24.31	-9.30	0.94	1.06	12.24

## Discussion

From 1990 to 2019, the US experienced an overall decrease in YLDs (33.07%), incidence (22.11%), and prevalence (29.93%) of bilateral LEAs. In addition, unilateral LEAs saw a decrease in YLDs (21.43%) and prevalence (15.61%) but an increase in incidence (9.05%) in the same time period. While there was an increase in the incidence of unilateral LEA, this could be due to patients who previously would have lost both lower limbs only having a unilateral amputation due to improved management of limb-threatening diseases and conditions.

LEAs have been linked to a variety of conditions, including vascular conditions, diabetes mellitus, trauma, malignancy, and congenital malformations [1]. Roughly 54% result from vascular disease, 45% result from trauma, and less than 2% result from cancer and other causes [15]. There is a constantly growing prevalence of diabetes mellitus around the world, leading to a higher prevalence of complications such as peripheral vascular disease [16]. However, studies suggest that despite higher rates of diabetic peripheral vascular disease, there is a decreasing rate of LEA in this population [17]. Improved pharmacotherapy, procedural care, and prevention strategies are a potential explanation for this trend. More broadly, this suggests an improvement in the management of diabetes and prevention of major complications. Improvements in diabetes care coordination and quality have driven improvements in risk factors for diabetes mellitus as well [17]. Lifestyle interventions such as diet and exercise are also increasingly being proven effective at improving glycemic control in diabetics, likely contributing to decreasing rates of complications [18].

Improvements in limb-sparing techniques and treatments are another potential source of contribution to decreased rates of amputations. In the time period of the study, there have been advances in antibiotic therapy and protocols for the treatment of infections that commonly lead to LEA, such as diabetic foot infections and necrotizing fasciitis [19,20]. In terms of limb-sparing tumor surgery, patients can keep their limbs while also enjoying improved clinical and oncologic outcomes [21]. Similarly, advances in surgical technology over the years have likely contributed to improved outcomes related to lower extremity trauma. Interestingly, the LEAP study, a large, prospective, observational study published in 2002, showed that two-year outcomes of patients who experienced severe leg injuries are typically equivalent between amputations and reconstruction [22]. Predictors of poorer outcomes included factors such as demographics, education level, insurance status, and smoking status [22].

Our analysis found that rates of YLDs, incidence, and prevalence were consistently higher in men compared to women. From 1990 to 2019, regardless of region, men had higher rates of both bilateral and unilateral LEAs in the US. This is consistent with the current data reported in the literature [15]. Furthermore, male sex is a risk factor for the development of peripheral vascular disease, diabetes mellitus is more prevalent in men, and rates of lower extremity trauma are higher in men [23-25]. All these factors together likely contribute to higher rates of lower limb loss in men compared to women.

Regional trends in YLDs, incidence, and prevalence rates of bilateral and unilateral LEAs are very similar over the studied time period. The regions having the highest and lowest rates of all three metrics are almost identical between bilateral and unilateral LEAs, with a few exceptions. Seeing that the trends in bilateral and unilateral LEAs match up consistently over time, it can be deduced that the factors local to each region are likely culprits for any regional discrepancies. These factors include, but are not limited to, things such as population, weather, urbanization, healthcare availability, resource allocation, and socioeconomic factors.

It should be noted that military and combat-related injuries are a distinct source of LEAs. Traumatic amputations related to explosive devices are a common cause of injury on the modern battlefield [26]. Figures 1-4C demonstrate distinct spikes in the incidence of bilateral and unilateral LEAs, which may be related to increased combat in warring regions where the US military is deployed. The heavy male skew of the US armed forces may also contribute to higher rates of LEAs among men [27]. Ultimately, service members who undergo LEAs as a result of combat-related injuries are burdened with significant disability as a result [28].

Doukas et al. present a study evaluating disability in patients undergoing LEA vs limb salvage after military-related trauma. While the study exclusively included US armed forces members, the results illustrate the significant disability faced by patients after LEA. Less than half of the patients at the time of the interview were participating in sports or recreational activities (45.1% unilateral LEA, 48.7% bilateral LEA) or working or back in active duty after their injuries (43.4% unilateral LEA, 30.8% bilateral LEA), demonstrating the functional disability that comes with LEAs. In terms of mental health, patients experienced depressive symptoms (40.7% unilateral LEA, 25.6% bilateral LEA), major depressive disorder (13.3% unilateral LEA, 10.3% bilateral LEA), and post-traumatic stress disorder (14.8% unilateral LEA, 10.3% bilateral LEA). Pain interferes with daily activity in a certain percentage of patients as well (17.1% unilateral LEA, 10.3% bilateral LEA). Interestingly, patients who underwent limb salvage surgery reported better functional scores but higher levels of disability across the board compared to those with an amputation [28]. In addition to disability after LEA, there are complications to the surgery that include surgical site infection, phantom limb pain, wound dehiscence, and repeat amputation [29].

Our study contains inherent limitations. First, this is a retrospective analysis, which is a limitation in and of itself. Our study was ecological in nature and, therefore, is limited by the ecological fallacy that population-level data apply to individuals, which is not the case. Data drawn from medical records can be unreliable because the data were not originally intended for research, so they can lack specific clinical granularity or glance over important aspects of a patient’s history. The data are also modeled, which can lend itself to uncertainty based on the simplification of the data. Since our data were drawn from the GBD dataset without inclusion or exclusion criteria, the limitations of the dataset are also inherent in our study. There is also a certain amount of bias in the results, bias in the fact that the data are often incomplete. Furthermore, results of retrospective studies can be over-generalized and should not necessarily be used to claim a cause-and-effect relationship [30].

Other significant limitations associated with this study are related to the GBD database. The database relies on the accuracy of medical coding, which can be inaccurate and can change over time. There may also be incomplete data due to certain hospital records not being included or patients’ data not being included for any reason. Additionally, factors that may be contributing to disease trends but are not accounted for in our analysis include, but are not limited to, socioeconomic status, comorbidities, and access to healthcare. All these factors may be confounders in the data that are not controlled for in our study.

## Conclusions

Overall, this broad-ranging temporal analysis of LEAs in the US from 1990 to 2019 has highlighted notable trends. In general, the US population experienced a significant drop in YLDs, prevalence, and incidence of both unilateral and bilateral LEAs. However, this promising development is still witnessed in the data in the setting of vast disparities between different genders and geographical locations. Throughout all regions, men have consistently experienced higher rates of YLDs, prevalence, and incidence of LEAs than women. The Western region had consistently high mean incidence and prevalence rates of LEAs while the Northeastern region repeatedly recorded the lowest rates.

The findings included in this study have demonstrated the significance of targeted public health interventions aimed at addressing the risk factors associated with LEAs, including diabetes mellitus, peripheral vascular diseases, neuropathy, and trauma. In addition, there are observed variations among sexes that affect overall LEA epidemiology. Additionally, subsequent research should focus on understanding why these differences exist and evaluating strategies for reducing them, especially for populations or regions with high vulnerability. Such data can be used by healthcare planners in making resource allocation decisions in the US concerning LEAs.
